# Social and Physical Environmental Factors Associated with Antihypertensive Medication Non-Adherence Among Older Adults with Hypertension in South Korea: Findings from the 2023 Community Health Survey

**DOI:** 10.3390/healthcare14182903

**Published:** 2026-09-08

**Authors:** Yunji Lee, Eunjoo Lee, Myo-Sung Kim

**Affiliations:** Department of Nursing, Research Institute of Dong-eui Nursing Science, Dong-eui University, Busan 47340, Republic of Korea; yjlee22@deu.ac.kr (Y.L.); ej.lee@deu.ac.kr (E.L.)

**Keywords:** hypertension, medication adherence, aged, social determinants of health, social participation, residence characteristics, health surveys

## Abstract

**Highlights:**

**What are the main findings?**

- Higher satisfaction with the community environment was independently associated with lower odds of antihypertensive medication non-adherence among older adults with hypertension in South Korea.
- Greater social participation was unexpectedly associated with higher odds of medication non-adherence, a finding that requires cautious and exploratory interpretation.

**What are the implications of the main findings?**

- Medication non-adherence among older adults with hypertension may be associated with perceived environmental and social characteristics as well as individual factors.
- Whether community-based approaches addressing perceived neighborhood conditions can improve medication adherence warrants evaluation in longitudinal or interventional studies.

**Abstract:**

**Background:** Hypertension requires lifelong antihypertensive medication to reduce cardiovascular risk. While most studies have focused on individual determinants of adherence, the role of perceived environmental and social characteristics remains unclear. This study examined the associations of depressive symptoms, satisfaction with the community environment, social participation, and social contact with antihypertensive medication non-adherence among older adults with hypertension in South Korea. **Methods:** A cross-sectional secondary analysis was conducted using the 2023 Korea Community Health Survey, including 44,822 adults aged 65 years and older with physician-diagnosed hypertension who were currently taking antihypertensive medication. Medication non-adherence was defined as taking medication on fewer than 24 of the previous 30 days, and associated factors were identified using complex sample multivariable logistic regression. **Results:** The prevalence of medication non-adherence was 0.4%. Higher satisfaction with the community environment was associated with lower odds of medication non-adherence (OR = 0.83, 95% CI = 0.72–0.95), whereas greater social participation was associated with higher odds (OR = 1.39, 95% CI = 1.09–1.78). Social contact, depressive symptoms, and the remaining characteristics were not significantly associated with medication non-adherence. **Conclusions:** Antihypertensive medication non-adherence among older adults is associated with perceived environmental characteristics as well as individual factors. Whether community-based approaches addressing these conditions can improve medication adherence requires evaluation in longitudinal or interventional studies.

## 1. Introduction

Hypertension is one of the most common chronic diseases and a major risk factor for serious cardiovascular and cerebrovascular diseases, including stroke and myocardial infarction. Maintaining blood pressure control through consistent adherence to antihypertensive medication is a cornerstone of hypertension management for preventing complications and reducing mortality [1]. As South Korea has entered a super-aged society, the prevalence of chronic diseases among adults aged 65 years and older continues to increase, and many of these individuals require lifelong antihypertensive therapy [2,3]. Medication adherence is regarded as one of the most cost-effective strategies for maximizing treatment outcomes while reducing healthcare costs. However, older adults are particularly vulnerable to medication non-adherence because of cognitive decline, polypharmacy, complex medication regimens, and financial burdens [1].

Previous studies have primarily focused on individual-level characteristics, such as age, sex, educational attainment, income level, disease-related knowledge, health beliefs, and medication side effects, as determinants of medication adherence among patients with hypertension [1,4]. More recently, however, public health and social epidemiology have increasingly emphasized that health behaviors are shaped not only by individual characteristics but also by the environments and social contexts in which people live. According to the socioecological model and the Social Determinants of Health framework, health behaviors are shaped by interactions between individual characteristics and the surrounding social and physical environments [5,6]. This perspective suggests that medication non-adherence among older adults with hypertension should be understood by considering perceived environmental characteristics and social relationships in addition to individual psychological characteristics.

Perceived environmental characteristics may provide an important foundation for helping older adults sustain self-care behaviors. Satisfaction with the community environment, including neighborhood trust, accessibility to healthcare services and public transportation, and community safety, may serve as a protective factor that supports healthy behaviors [7]. Likewise, social participation and social contact have been shown to reduce social isolation while facilitating access to health-related information and supporting the maintenance of healthy behaviors [1,8]. Despite these potential benefits, evidence regarding the associations between social and physical environmental factors and medication non-adherence among older adults with hypertension remains limited. Moreover, few studies have simultaneously considered individual psychological factors together with social and physical environmental characteristics.

Depressive symptoms have frequently been identified as an individual-level factor associated with poor medication adherence among older adults with chronic diseases. Late-life depression may reduce motivation, increase feelings of lethargy, and impair self-care capacity, and has therefore been linked to lower adherence to prescribed medications [9]. Psychological factors such as depressive symptoms may also operate alongside environmental conditions in shaping health behaviors [4]. Understanding medication non-adherence among older adults with hypertension therefore requires an approach that considers individual psychological characteristics together with social and physical environmental factors, including satisfaction with the community environment, social participation, and social contact.

Although the acute phase of the COVID-19 pandemic has ended, the reduction in social activities, changes in healthcare utilization, and mental health challenges experienced during the pandemic may continue to relate to chronic disease management among older adults [10]. The pandemic substantially reduced older adults’ social contacts and participation in community activities while altering perceptions of their community environments, and these changes may continue to affect their daily lives and chronic disease management [11]. In chronic conditions such as hypertension, which require lifelong medication use, it is important to understand how social networks and perceived environmental conditions relate to medication non-adherence. In this context, the 2023 Korea Community Health Survey (KCHS) provides data collected during the early stage of post-pandemic recovery. It should be noted that this study does not aim to evaluate the effects of the COVID-19 pandemic, as it neither measured COVID-19 exposure nor compared pre- and post-pandemic periods; the post-pandemic period is referred to as the context in which the data were collected rather than as a factor examined in this study.

Therefore, this study used data from the 2023 KCHS to examine the prevalence of medication non-adherence among South Korean adults aged 65 years and older with hypertension. Based on a socioecological perspective, we investigated the associations of an individual-level factor (depression) and social and environmental factors (satisfaction with the community environment, social participation, and social contact) with medication non-adherence. The findings are expected to provide evidence for understanding medication non-adherence among older adults with hypertension from a broader perspective that incorporates both individual characteristics and social and physical environmental factors. Furthermore, this study may contribute to the development of community-based chronic disease management strategies and tailored intervention programs for older adults with hypertension.

## 2. Materials and Methods

### 2.1. Study Design

This study employed a cross-sectional secondary data analysis design to examine the association between antihypertensive medication non-adherence and socio–physical environmental factors among older adults aged 65 years and older in South Korea.

### 2.2. Study Data and Participants

This study used data from the 2023 KCHS, which is conducted annually by the Korea Disease Control and Prevention Agency (KDCA) in accordance with Article 4 of the Regional Public Health Act. The target population of the KCHS comprises adults aged 19 years and older residing in South Korea. Sample areas were selected using probability proportional-to-size systematic sampling based on administrative districts, and sample households were subsequently selected using systematic sampling. Between 16 May and 31 July 2023, trained interviewers visited selected households and conducted face-to-face household and individual surveys using tablet PC-based electronic questionnaires [12].

**Figure 1:**
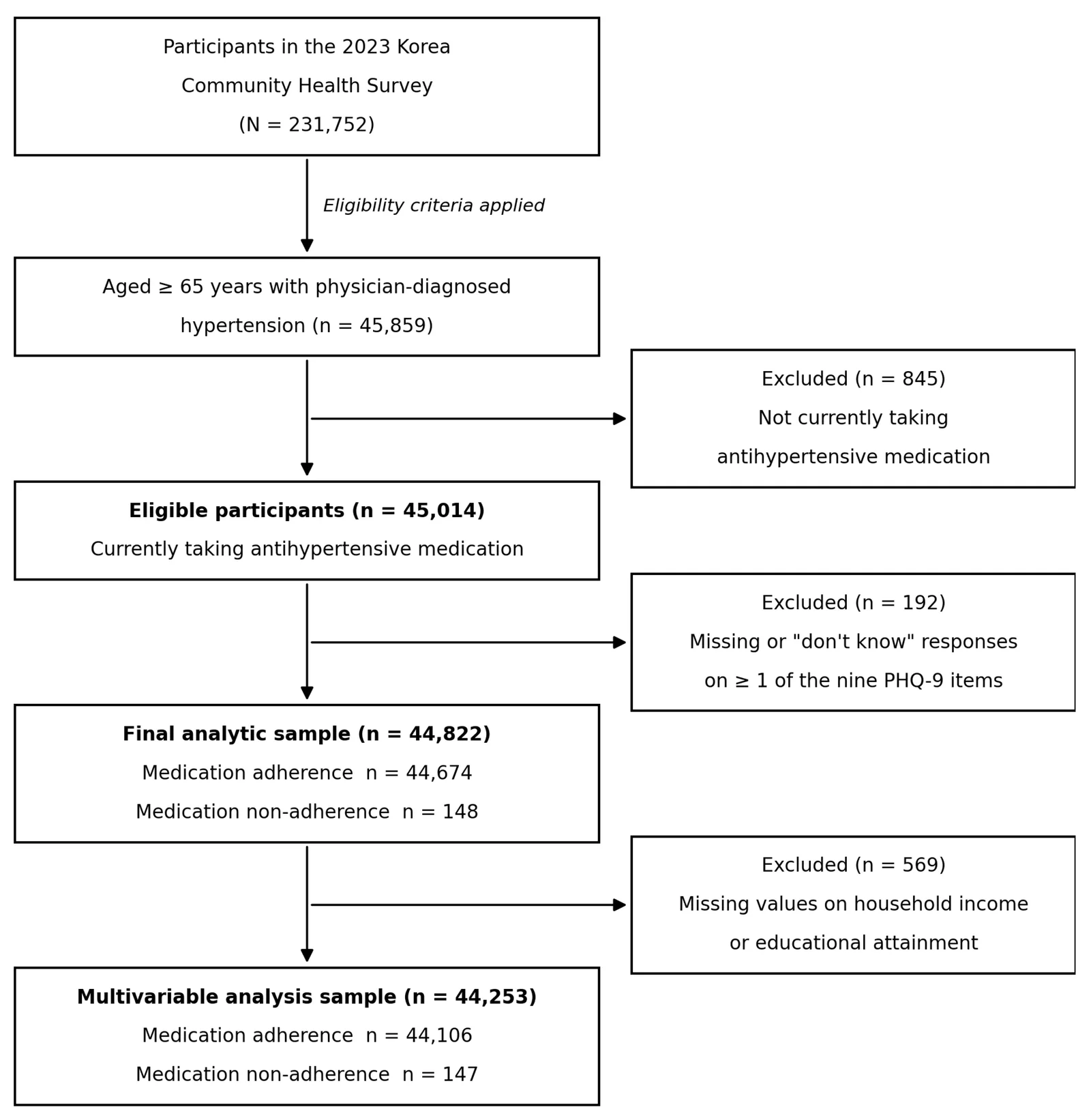
Flow diagram of participant selection. PHQ-9, Patient Health Questionnaire-9. The multivariable analysis sample refers to participants with complete data on all covariates included in the adjusted regression model.

To examine the association between antihypertensive medication non-adherence and socio–physical environmental factors among older adults, data from the 2023 KCHS were used because they included relevant socio–physical environmental variables. Among the 231,752 participants in the 2023 KCHS, we first applied the eligibility criteria: (1) aged 65 years or older, (2) diagnosed with hypertension by a physician, and (3) currently taking antihypertensive medication. Of the 45,014 participants who met these criteria, 192 (0.4%) were excluded because of missing or “don’t know” responses on one or more of the nine PHQ-9 items, as the total score could not be calculated, yielding a final analytic sample of 44,822. The participant selection process is summarized in Figure 1.

Among the 44,822 participants, missing values were present for household income (*n* = 555, 1.2%) and educational attainment (*n* = 19, 0.04%); all other study variables were complete. Because the proportion of missing values was small and confined to two covariates, all 44,822 participants were retained for the descriptive analyses, with percentages based on available cases for each variable.

Because eligibility required current use of antihypertensive medication, individuals who had entirely discontinued prescribed therapy were not captured in the analytic population. The KCHS does not distinguish between respondents who had never been prescribed antihypertensive medication and those who had discontinued it, so non-adherence could not be determined for participants managing their blood pressure through non-pharmacological measures alone. The estimand of this study is therefore non-adherence among current users of antihypertensive medication, rather than non-adherence among all older adults with diagnosed hypertension. This group accounted for 1.8% of participants with diagnosed hypertension. Because individuals who have discontinued therapy may represent more severe non-adherence, this restriction is likely to have biased the observed prevalence downward.

### 2.3. Study Variables

#### 2.3.1. Antihypertensive Medication Non-Adherence

Antihypertensive medication non-adherence was determined using the KCHS items on hypertension treatment. Participants who reported a physician diagnosis of hypertension were asked whether they were currently receiving treatment to control their blood pressure, including whether they were taking antihypertensive medication (“Taking blood pressure medication: yes/no”; item hya_14a2). Those who answered affirmatively were then asked on how many days during the previous month they had taken it (“How many days per month?”; item hya_14b2). Based on the widely accepted 80% adherence threshold used in medication adherence studies [13,14], the denominator was standardized to 30 days: participants who took their medication on ≥24 days during the previous month were classified as the medication adherence group, whereas those who took it on <24 days were classified as the medication non-adherence group. The KCHS item refers to antihypertensive medication in general rather than to specific agents and records the number of days on which medication was taken rather than the number of doses taken per day; adherence was therefore assessed at the level of days of use within a 30-day reference period.

#### 2.3.2. Sociodemographic Characteristics

Sociodemographic characteristics included sex, age, region of residence, household type, educational attainment, and income level. Region of residence was classified into urban and rural areas according to administrative districts. Household type was categorized as single-person households or multi-person households.

Educational attainment was classified according to the highest level of education completed. In the KCHS, education is recorded as a combination of school level and enrolment status (currently enrolled, on leave of absence, withdrawn, or graduated), and the KCHS guidelines do not specify how these responses should be collapsed into a single measure of attainment. We therefore applied the classification rule specified in the raw data use guidelines of the Korea National Health and Nutrition Examination Survey, issued by the same agency [15], under which respondents who had not completed their current level of schooling, including those currently enrolled, on leave of absence, or having withdrawn, are assigned to their highest completed level of education. Educational attainment was categorized into four groups: elementary school graduation or lower, middle school graduation, high school graduation, and college graduation or higher.

Income level was determined using equivalized monthly household income. For participants who reported an exact income amount, the reported value was used; for those who reported an income category, the midpoint of the category was used. Monthly household income was divided by the square root of household size to obtain equivalized income, which corresponds to the income of a single-person household. Equivalized income was then compared with the 2023 standard median income for a single-person household announced by the Ministry of Health and Welfare (2,077,892 KRW per month) [16], and income level was categorized into four groups: low (<50% of the median), mid-low (≥50% to <100%), mid-high (≥100% to <150%), and high (≥150%).

#### 2.3.3. Health-Related Characteristics

Health-related characteristics included comorbid diabetes mellitus, subjective health status, and depressive symptoms. Comorbidity was assessed using physician-diagnosed diabetes mellitus, coded as present or absent. Because all participants had hypertension by design, diabetes was the only additional chronic condition available in the 2023 KCHS that could be included as a covariate.

Subjective health status was categorized into five levels: very good, good, fair, poor, and very poor.

Depressive symptoms were assessed using the Korean version of the Patient Health Questionnaire-9 (PHQ-9) [17,18]. The PHQ-9 consists of nine items corresponding to the diagnostic criteria for major depressive disorder. Each item is rated on a 4-point Likert scale ranging from 0 to 3, with total scores ranging from 0 to 27. Based on total scores, depressive symptoms were categorized as normal (0–4), mild (5–9), moderate (10–14), moderately severe (15–19), and severe (≥20). A total score of ≥10 was defined as the presence of depressive symptoms [17,18]. Previous studies using the Korean version of the PHQ-9 among older adults reported a Cronbach’s α of 0.86 [18], and Cronbach’s α was 0.79 in the present study.

#### 2.3.4. Socio–Physical Environmental Factors

Socio–physical environmental factors included satisfaction with the community environment, social participation, and social contact. All three measures are individual-level self-reported assessments. The community-environment measure captures respondents’ subjective perceptions of their residential surroundings rather than objectively measured contextual attributes, and no neighborhood-level aggregation or multilevel modeling was performed.

Satisfaction with the community environment was measured using seven items assessing participants’ perceptions of their residential community: trust among neighbors, availability of assistance from neighbors during family or ceremonial events, community safety, natural environment, living environment, public transportation, and satisfaction with healthcare services. Each item was assessed using a binary response format (“yes” or “no”). Refusals were negligible across all seven items (≤0.04% each), whereas “don’t know” responses ranged from 0.3% (natural and living environment) to 2.1% (trust among neighbors); 1837 participants (4.1%) had a refusal or “don’t know” response on at least one item. Responses of “yes” were assigned 1 point, whereas responses of “no”, refusals, and “don’t know” were assigned 0 points, on the assumption that a non-affirmative response reflects the absence of a perceived positive environmental attribute. Because “don’t know” responses may indicate an inability to evaluate the attribute rather than a negative evaluation, a sensitivity analysis restricted to the 42,985 participants (95.9%) who provided a substantive response to all seven items was conducted. The scores of the seven items were summed, with total scores ranging from 0 to 7. Higher scores indicated greater satisfaction with the community environment.

Social participation was measured by summing participation in four types of activities: religious activities, social/fellowship activities, leisure or recreational activities, and charitable organization activities. Participants who reported regularly participating in each activity at least once a month were assigned 1 point for each activity. Total scores ranged from 0 to 4, with higher scores indicating greater levels of social participation. The four activity types were combined into a single count score to represent the overall breadth of engagement in community-based activities. Because the activities differ in purpose and setting, each activity type was additionally examined separately (Supplementary Table S1).

Social contact was assessed using three items measuring the frequency of contact with relatives, neighbors, and friends. Regular contact was defined as meeting or communicating at least once a week. Each item was coded as 1 for regular contact and 0 otherwise. The three items were summed, resulting in total scores ranging from 0 to 3. Higher scores indicated more frequent social contact.

The three composite scores are investigator-constructed indices derived from individual KCHS items rather than previously validated scales. Internal consistency in the present sample was modest (KR-20 = 0.56 for satisfaction with the community environment, 0.40 for social participation, and 0.37 for social contact). These measures are formative rather than reflective indices: the constituent items represent distinct facets of the residential and social environment that are not expected to covary strongly, and internal consistency is therefore not an appropriate criterion for their validity. The absence of prior validation is acknowledged as a limitation.

### 2.4. Ethical Considerations

This study was conducted after obtaining an exemption from Institutional Review Board review (DIRB-202607-HR-W-25) from Dong-eui University. An application to access the raw data was submitted through the KCHS website (https://chs.kdca.go.kr), and the data were downloaded and analyzed according to the data access procedures [19]. The dataset contained no personally identifiable information, and participant anonymity was maintained. This study complied with the guidelines for the use of raw data provided by the Korea Disease Control and Prevention Agency.

### 2.5. Statistical Analysis

All analyses accounted for the complex sampling design of the KCHS. A complex sample analysis plan was created in SPSS using the stratification variable (kstrata), the cluster variable (SPOT_NO), and the sampling weight (wt_p) specified in the KCHS raw data use guidelines [12], assuming with-replacement sampling. Estimation was restricted to the eligible subpopulation—adults aged ≥65 years with physician-diagnosed hypertension who were currently taking antihypertensive medication—using the subpopulation specification within each complex samples procedure, so that variance estimation retained the full sample design information.

Statistical analyses were performed using SPSS/WIN version 29.0 (IBM Corp., Armonk, NY, USA). All statistical tests were two-sided, and statistical significance was set at *p* < 0.05. The specific analytical procedures were as follows:

1. Participants’ sociodemographic characteristics, health-related characteristics, and socio–physical environmental factors were summarized using unweighted frequencies, weighted percentages, means, and standard errors.
2. Differences according to medication adherence status were examined using design-based Wald F tests from complex sample logistic regression models fitted separately for each categorical characteristic, and complex sample general linear models for continuous variables.
3. Complex sample logistic regression analysis was conducted to identify factors associated with antihypertensive medication non-adherence. Multicollinearity among independent variables was assessed using tolerance and variance inflation factor values. Linearity in the logit was examined by comparing models in which each socio–physical environmental score was entered as a continuous variable with models in which it was entered as a categorical variable. For social participation and social contact, the category- specific log-odds followed a monotonic pattern consistent with a linear specification. For satisfaction with the community environment, the pattern was broadly consistent in the upper score range, but estimates for the lowest categories were unstable because of very small cell counts. The scores were therefore retained as continuous predictors.

Regression models were fitted using a complete-case approach; 569 participants (1.3%) with a missing value on at least one covariate were excluded from Model II. Covariates in Model II were selected on the basis of the socioecological framework guiding this study, comprising sociodemographic and health-related characteristics at the individual level. For the regression models, educational attainment was dichotomized as middle school graduation or lower versus high school graduation or higher; household income as below versus at or above the standard median income for a single-person household; self-rated health as good (very good or good) versus poor (fair, poor, or very poor); and depressive symptoms using the PHQ-9 cut-off of ≥10 [17,18]. These collapsing rules were based on the expected number of non-adherence events rather than on observed associations. With 147 non-adherence events and 13 predictor terms in Model II, the events-per-variable ratio was 11.3, exceeding the conventional minimum of 10 recommended for logistic regression [20]. Two sensitivity analyses were conducted: one restricted to participants who provided a substantive response to all seven community-environment items, and one using an alternative 90% adherence threshold (<27 days). In addition, the four types of social participation were modeled separately in place of the composite score.

## 3. Results

### 3.1. Differences in Sociodemographic and Health-Related Characteristics According to Medication Non-Adherence

Among the 44,822 participants included in this study, 44,674 (99.6%) were classified into the medication adherence group and 148 (0.4%) into the medication non-adherence group. Among all participants, 57.0% were female, and the mean age was 74.7 years (SE = 0.05). Participants aged 65–74 years accounted for the largest proportion (52.4%). Most participants lived in urban areas (72.0%), lived in multi-person households (76.9%), and had an educational attainment of elementary school graduation or lower (47.1%). Regarding income level, 39.9% were classified as low income and 31.9% as mid-low income, indicating that approximately 72% of participants had income levels below the median income level.

Regarding subjective health status, the most common category was fair (41.0%), followed by poor (30.0%). Comorbid diabetes mellitus was reported by 30.9% of participants. For depressive symptoms, 78.2% of participants were classified as normal, and 6.0% screened positive for depressive symptoms based on a PHQ-9 score of ≥10 (Table 1).

The differences in sociodemographic and health-related characteristics according to medication non-adherence status are presented in Table 1. No statistically significant difference was observed between the two groups in any of the characteristics examined, including sex, age, region of residence, household type, educational attainment, household income, comorbid diabetes, subjective health status, or depressive symptoms. The mean PHQ-9 score was somewhat higher in the medication non-adherence group (3.74, SE = 0.53) than in the medication adherence group (2.90, SE = 0.02), but this difference was not statistically significant (F = 2.498, *p* = 0.114). Likewise, the proportion screening positive for depressive symptoms did not differ significantly between the non-adherence (9.6%) and adherence (6.0%) groups (F = 1.696, *p* = 0.148).

### 3.2. Differences in Socio–Physical Environmental Factors According to Medication Non-Adherence

The differences in socio–physical environmental factors according to medication non-adherence status are presented in Table 2.

The mean score of satisfaction with the community environment was significantly higher in the medication adherence group (5.49, SE = 0.01) than in the medication non-adherence group (5.06, SE = 0.18) (F = 5.871, *p* = 0.015). In contrast, the mean social participation score did not differ significantly between the two groups (F = 3.701, *p* = 0.054), although it was somewhat higher in the medication non-adherence group (1.35, SE = 0.14) than in the medication adherence group (1.08, SE = 0.01). No significant difference was observed in the mean social contact score between the medication adherence (1.63, SE = 0.01) and non-adherence (1.48, SE = 0.11) groups (F = 1.777, *p* = 0.183).

### 3.3. Association Between Socio–Physical Environmental Factors and Medication Non-Adherence

Complex sample multivariable logistic regression analysis was conducted to examine the associations between socio–physical environmental factors and antihypertensive medication non-adherence. Two models were constructed. Model I included only the three socio–physical environmental factors (*n* = 44,822, non-adherence events = 148), whereas Model II additionally adjusted for sociodemographic and health-related characteristics (*n* = 44,253, non-adherence events = 147) (Table 3). Both models converged without difficulty, and the parameter estimates showed no evidence of instability. The assessment of multicollinearity among independent variables showed that variance inflation factor (VIF) values ranged from 1.041 to 1.314, indicating no evidence of multicollinearity among the variables included in the regression models.

In Model I, higher satisfaction with the community environment was associated with lower odds of medication non-adherence (OR = 0.83, 95% CI = 0.72–0.96). Conversely, higher social participation was associated with higher odds of medication non-adherence (OR = 1.34, 95% CI = 1.07–1.69).

In Model II, after adjusting for sociodemographic and health-related characteristics, satisfaction with the community environment remained associated with lower odds of medication non-adherence (OR = 0.83, 95% CI = 0.72–0.95). Social participation remained associated with higher odds of medication non-adherence (OR = 1.39, 95% CI = 1.09–1.78). Social contact and the remaining sociodemographic and health-related characteristics were not significantly associated with medication non-adherence. Because non-adherence was rare (0.4%), these odds ratios are interpreted as changes in the odds of non-adherence and should not be read as changes in absolute risk.

### 3.4. Sensitivity Analyses

Two sensitivity analyses were conducted (Supplementary Table S2). First, the analysis was repeated among participants who provided a substantive response to all seven community-environment items (*n* = 42,546, non-adherence events = 141). The weighted prevalence of non-adherence was unchanged (0.4%). The associations for satisfaction with the community environment (aOR = 0.82, 95% CI = 0.71–0.95) and social participation (aOR = 1.39, 95% CI = 1.08–1.80) were consistent with the primary analysis. The association for social contact reached statistical significance in this restricted sample (aOR = 0.80, 95% CI = 0.65–0.97), although the point estimate was similar to that of the primary analysis (aOR = 0.85, 95% CI = 0.69–1.04).

Second, medication non-adherence was redefined using a 90% threshold (<27 days during the previous month), which yielded 160 non-adherence events (*n* = 44,253). The estimates were essentially unchanged from the primary analysis for satisfaction with the community environment (aOR = 0.83, 95% CI = 0.73–0.94), social participation (aOR = 1.39, 95% CI = 1.11–1.75), and social contact (aOR = 0.85, 95% CI = 0.70–1.03).

In an additional exploratory, the four types of social participation were entered separately in place of the composite score, with all other covariates identical to Model II. Only leisure or recreational activities were associated with medication non-adherence (aOR = 2.35, 95% CI = 1.46–3.80); the associations for religious (aOR = 1.27, 95% CI = 0.77–2.09), social or fellowship (aOR = 0.97, 95% CI = 0.56–1.69), and charitable organization activities (aOR = 1.29, 95% CI = 0.58–2.88) were not statistically significant (Supplementary Table S1). Because this analysis involved multiple activity-specific comparisons without adjustment for multiplicity, the result should be regarded as exploratory rather than as a confirmed activity-specific effect.

## 4. Discussion

This study examined the associations between antihypertensive medication non-adherence and socio–physical environmental factors among older adults with hypertension in South Korea using data from the 2023 KCHS. In particular, by simultaneously considering depressive symptoms as an individual-level factor and satisfaction with the community environment, social participation, and social contact as social and environmental factors, this study sought to understand medication non-adherence from a socioecological perspective that incorporates both individual characteristics and perceived environmental conditions [5]. Higher satisfaction with the community environment was independently associated with lower odds of medication non-adherence, whereas higher social participation was associated with higher odds. Social contact and depressive symptoms were not significantly associated with medication non-adherence in either the univariate or the multivariable analyses.

First, the medication adherence rate in this study was 99.6%, and the non-adherence rate was 0.4%. This rate is considerably higher than those reported in previous domestic and international studies [8,21]. This finding should be interpreted cautiously because the KCHS assesses medication-taking behavior during the previous month using self-reported questionnaires, which may be influenced by social desirability bias and recall bias [22]. In addition, because medication non-adherence was defined as taking prescribed antihypertensive medication on fewer than 80% of days during the previous month, intermittent medication omissions or long-term adherence patterns may not have been fully captured [8]. Eligibility also required current use of antihypertensive medication, so individuals who had entirely discontinued prescribed therapy were not captured in the analytic population. Therefore, the findings of this study should be interpreted primarily in terms of the direction and relative magnitude of associations between medication non-adherence and related factors rather than the absolute prevalence of non-adherence.

In this study, depressive symptoms were not significantly associated with antihypertensive medication non-adherence in either the univariate or the multivariable analysis. Although the mean PHQ-9 score and the proportion screening positive for depressive symptoms were somewhat higher in the non-adherence group, neither difference reached statistical significance. This finding differs from previous studies reporting that late-life depression is associated with poorer medication adherence among older adults with chronic conditions [9,23,24,25].

Several explanations are possible. The very low prevalence of non-adherence in this sample (0.4%) limited the statistical power to detect associations for any individual characteristic, and no sociodemographic or health-related factor was significantly associated with non-adherence in this study. In addition, the PHQ-9 captures depressive symptoms over the preceding two weeks, whereas medication-taking behavior was assessed over the previous month; the two measures may therefore not correspond to the same period. Because this study did not examine interactions or mediating pathways between depressive symptoms and socio-environmental factors, the absence of an association should not be taken as evidence that depressive symptoms are unrelated to medication-taking behavior. Longitudinal studies with clinical assessment of depression are needed to clarify the relationships among depressive symptoms, social support, community environments, and medication adherence.

Higher satisfaction with the community environment was independently associated with lower odds of medication non-adherence in this study. This finding is consistent with the perspectives of social determinants of health and socioecological models, which emphasize that health behaviors are shaped not only by individual characteristics but also by the environments and resources surrounding individuals [5,26]. One possible interpretation is that positive perceptions of neighborhood trust, safety, accessibility to healthcare services, and transportation reflect an environmental context that supports healthcare utilization and access to health-related information [27,28,29]. However, none of these pathways were directly measured in this study, and this interpretation should be regarded as a hypothesis requiring empirical examination. Previous studies have demonstrated that community environments are associated with physical activity, healthcare utilization, and chronic disease management [6]. The present study extends this evidence by suggesting that perceived environmental characteristics may also be relevant to medication non-adherence among older adults with hypertension.

The magnitude of this association should be interpreted in context. A one-point increase in the seven-item community-environment score corresponded to 17% lower odds of non-adherence, which, given the low baseline prevalence (0.4%), translates into an absolute difference of less than one percentage point. These findings therefore indicate a consistent direction of association rather than a large difference in the absolute frequency of non-adherence. Whether hypertension management strategies that address perceived environmental conditions alongside individual education and counseling can improve medication adherence warrants evaluation in longitudinal or interventional studies.

Conversely, higher social participation was associated with higher odds of medication non-adherence, which was contrary to expectations. Social participation is generally considered a protective factor that promotes social support and healthy behaviors [30,31]. This unexpected finding should be regarded as exploratory for several reasons. First, the mean social participation score did not differ significantly between the adherence and non-adherence groups (F = 3.701, *p* = 0.054), and reached statistical significance only in the multivariable model, indicating that it was not robust across analyses. Second, because of the cross-sectional design, the direction of the relationship cannot be determined; older adults who are functionally able to participate in social activities may differ systematically from those who are not, in ways that also relate to medication-taking behavior. Third, unmeasured factors such as health behaviors, daily routines, or functional status may confound this association [1].

The composite social participation score also masked substantial heterogeneity across activity types [32]. In an exploratory analysis in which the four activities were modeled separately, the association was confined to leisure or recreational activities (aOR = 2.35, 95% CI = 1.46–3.80), whereas religious, fellowship, and charitable activities showed no significant associations. Because this analysis involved multiple activity-specific comparisons, this pattern should not be interpreted as a confirmed activity-specific effect; it does, however, suggest that the composite score does not reflect a uniform effect of social engagement. One possible interpretation concerns differences in the social contexts in which these activities may take place. Religious, fellowship, and charitable activities may involve more established social groups, whereas leisure and recreational activities may occur in more varied social settings. Previous qualitative work has reported that some older adults are reluctant to be seen as ill and omit medications in social settings [33]. This tendency may be relevant to the present finding, particularly in the leisure and recreational settings described above. However, neither the social context of activities nor medication-taking routines were measured in this study, and this interpretation remains a hypothesis. Studies examining the type, purpose, duration, and social context of activities, together with direct measurement of daily medication-taking behavior, are needed before any behavioral mechanism can be established.

Social contact was not significantly associated with medication non-adherence in the primary analysis, although the point estimate suggested a direction consistent with a protective association (aOR = 0.85, 95% CI = 0.69–1.04). In the sensitivity analysis restricted to participants with complete responses on the community-environment items, this association reached statistical significance (aOR = 0.80, 95% CI = 0.65–0.97), with a point estimate similar to that of the primary analysis. Given that the two estimates differ little in magnitude and that the confidence interval in the restricted sample only marginally excluded 1, this finding warrants cautious interpretation.

One possible explanation for the absence of a clear association is that the frequency of social contact alone may not adequately capture the functional or qualitative aspects of social support relevant to medication-taking behavior [34,35]. Previous studies have suggested that practical and emotional forms of social support are associated with medication adherence, highlighting the importance of the functional aspects of social support rather than social contact alone [34]. Therefore, future studies should consider not only the frequency of social contact but also its quality and functional aspects.

This study utilized data collected during the early post-pandemic recovery period, thereby reflecting the social environment during that phase. However, because this study neither directly examined the effects of the COVID-19 pandemic nor compared pre- and post-pandemic periods, the findings should be interpreted within the broader context of the post-pandemic recovery period.

A major strength of this study is the use of nationally representative data with a survey-weighted analytical framework to examine medication non-adherence among older adults with hypertension, considering individual psychological characteristics together with perceived socio–physical environmental factors. However, several limitations should be noted. First, the cross-sectional design precludes causal inference [36]. Second, medication-taking behavior was self-reported over a one-month period, which may have overestimated adherence and may not reflect long-term patterns [8,22]; because eligibility required current medication use, individuals who had discontinued therapy were also not captured. Third, the KCHS does not record the number of prescribed medications, duration of hypertension or treatment, adverse effects, or healthcare utilization, all of which remain potential sources of residual confounding. Fourth, the socio–physical environmental measures were investigator-constructed indices without prior validation, based on participants’ perceptions rather than objective community characteristics, and did not capture the qualitative aspects of social participation or social support; because these indices were specified as formative measures, conventional internal consistency statistics are not directly applicable [37]. Fifth, missing and “don’t know” responses on the community-environment items were coded as 0, which may introduce exposure misclassification, although a sensitivity analysis restricted to complete responders yielded consistent results. Finally, linearity in the logit could not be fully verified for satisfaction with the community environment because of sparse data in the lowest score categories.

Future studies should employ longitudinal designs and objective measures of medication adherence to clarify the pathways linking individual psychological characteristics and perceived environmental conditions to medication-taking behavior. Multilevel studies incorporating objectively measured community characteristics and intervention trials are also warranted.

## 5. Conclusions

This study examined the associations between medication non-adherence and socio–physical environmental factors among older adults with hypertension in South Korea using data from the 2023 KCHS. Higher satisfaction with the community environment was independently associated with lower odds of medication non-adherence, whereas greater social participation was associated with higher odds. Depressive symptoms and social contact were not significantly associated with medication non-adherence.

These findings suggest that medication non-adherence among older adults with hypertension was associated with perceived social and environmental characteristics as well as individual factors. They support considering perceived social and environmental conditions, alongside individual characteristics, when developing strategies to improve medication adherence, although the unexpected association with social participation should be regarded as exploratory.

Because this study used a cross-sectional design and self-reported data, causal inferences cannot be drawn. Longitudinal studies using objective measures of medication adherence are needed to clarify the pathways linking perceived environmental conditions and medication-taking behavior, and to evaluate whether interventions addressing both individual and environmental factors can improve adherence.

## Figures and Tables

**Table 1:** Differences in sociodemographic and health-related characteristics according to medication non-adherence.

Characteristics	Categories	Overall (*n* = 44,822)	Medication		
			Adherence (*n* = 44,674)	Non-Adherence (*n* = 148)	F (*p*)
		*n* ^†^ (Weighted%) ^‡^	*n* ^†^ (Weighted%) ^‡^		
Sex	Male	17,749 (43.0)	17,692 (43.0)	57 (36.9)	1.318 (0.251)
	Female	27,073 (57.0)	26,982 (57.0)	91 (63.1)	
Age (years)	65–74	21,680 (52.4)	21,601 (52.4)	79 (49.7)	0.158 (0.853)
	75–84	17,992 (38.1)	17,939 (38.1)	53 (41.2)	
	≥85	5150 (9.5)	5134 (9.5)	16 (9.1)	
	Mean ± SE ^‡^	74.74 ± 0.05	74.74 ± 0.05	74.74 ± 0.84	0.000 (0.998)
Region of residence	Urban	18,551 (72.0)	18,492 (72.0)	59 (72.9)	0.038 (0.846)
	Rural	26,271 (28.0)	26,182 (28.0)	89 (27.1)	
Household type	Single-person	12,906 (23.1)	12,871 (23.2)	35 (16.9)	2.359 (0.125)
	Multi-person	31,916 (76.9)	31,803 (76.8)	113 (83.1)	
Educational attainment	≤Elementary school	25,886 (47.1)	25,811 (47.1)	75 (41.1)	1.591 (0.189)
	Middle school	7631 (18.7)	7606 (18.7)	25 (20.6)	
	High school	7923 (22.7)	7894 (22.8)	29 (19.0)	
	≥College	3363 (11.5)	3344 (11.4)	19 (19.4)	
Household income	Low	21,681 (39.9)	21,612 (39.9)	69 (43.6)	1.150 (0.327)
	Middle-low	13,178 (31.9)	13,137 (31.9)	41 (25.4)	
	Middle-high	6199 (17.5)	6170 (17.4)	29 (23.5)	
	High	3209 (10.7)	3201 (10.7)	8 (7.4)	
Comorbid diabetes mellitus	Yes	13,657 (30.9)	13,626 (30.9)	31 (22.9)	1.934 (0.164)
	No	31,165 (69.1)	31,048 (69.1)	117 (77.1)	
Subjective health status	Very good	637 (1.6)	635 (1.6)	2 (0.5)	1.092 (0.359)
	Good	7694 (19.1)	7666 (19.1)	28 (16.3)	
	Fair	17,545 (41.0)	17,476 (40.9)	69 (47.9)	
	Poor	14,706 (30.0)	14,669 (30.0)	37 (25.4)	
	Very poor	4240 (8.4)	4228 (8.4)	12 (9.9)	
Depressive symptoms	Normal	35,675 (78.2)	35,559 (78.2)	116 (70.8)	1.696 (0.148)
	Mild	6775 (15.8)	6754 (15.8)	21 (19.5)	
	Moderate	1595 (4.1)	1590 (4.1)	5 (3.2)	
	Moderately severe	560 (1.4)	555 (1.4)	5 (5.0)	
	Severe	217 (0.5)	216 (0.5)	1 (1.4)	
	Mean ± SE ^‡^	2.90 ± 0.02	2.90 ± 0.02	3.74 ± 0.53	2.498 (0.114)

SE = standard error. ^†^ Unweighted frequencies; ^‡^ Weighted estimates. Analyses were based on available cases for each variable: educational attainment (*n* = 44,803) and household income (*n* = 44,267); all other variables had complete data. Design-based Wald F statistics were obtained from complex sample logistic regression models fitted separately for each categorical characteristic, and from complex sample general linear models for continuous variables; numerator degrees of freedom varied by the number of categories, with denominator df = 15,377.

**Table 2:** Differences in socio–physical environmental factors according to medication non-adherence.

Variables	Overall (*n* = 44,822)	Medication			
		Adherence (*n* = 44,674)	Non-Adherence (*n* = 148)	F	*p*
	*n* ^†^ (Weighted% ^‡^)	*n* ^†^ (Weighted% ^‡^)			
Satisfaction with the community environment					
0	221 (0.6)	221 (0.6)	0 (0.0)		
1	318 (0.9)	314 (0.9)	4 (4.7)		
2	871 (2.3)	866 (2.3)	5 (1.1)		
3	2016 (5.5)	2005 (5.5)	11 (13.1)		
4	4173 (11.0)	4153 (11.0)	20 (11.5)		
5	9555 (23.8)	9520 (23.8)	35 (27.9)		
6	10,763 (27.1)	10,739 (27.1)	24 (17.8)		
7	16,905 (28.8)	16,856 (28.8)	49 (24.0)		
M ± SE ^‡^	5.49 ± 0.01	5.49 ± 0.01	5.06 ± 0.18	5.871	0.015
Social participation					
0	16,546 (33.4)	16,498 (33.4)	48 (28.6)		
1	16,598 (36.4)	16,551 (36.4)	47 (29.8)		
2	8415 (20.9)	8379 (20.9)	36 (26.5)		
3	2573 (7.3)	2564 (7.3)	9 (8.1)		
4	690 (2.0)	682 (2.0)	8 (6.9)		
M ± SE ^‡^	1.08 ± 0.01	1.08 ± 0.01	1.35 ± 0.14	3.701	0.054
Frequency of social contact					
0	4546 (15.7)	4526 (15.6)	20 (18.0)		
1	10,373 (28.3)	10,340 (28.3)	33 (34.3)		
2	17,096 (33.6)	17,046 (33.6)	50 (29.1)		
3	12,807 (22.4)	12,762 (22.5)	45 (18.7)		
M ± SE ^‡^	1.63 ± 0.01	1.63 ± 0.01	1.48 ± 0.11	1.777	0.183

SE = standard error. ^†^ Unweighted frequencies; ^‡^ Weighted estimates. Group comparisons were based on mean scores using complex sample general linear models with design-based Wald F statistics (df = 1, 15,377). Because the three measures are ordinal summary scores analyzed as continuous variables, category-level distributions are presented descriptively without separate significance testing.

**Table 3:** Association between socio–physical environmental factors and medication non-adherence.

Variables		Medication Non-Adherence (OR (95% CI)) ^†^	
		Model I (*n* = 44,822)	Model II (*n* = 44,253)
Satisfaction with the community environment		0.83 ** (0.72–0.96)	0.83 ** (0.72–0.95)
Social participation		1.34 ** (1.07–1.69)	1.39 ** (1.09–1.78)
Frequency of social contact		0.87 (0.70–1.07)	0.85 (0.69–1.04)
Sex	Men (Ref)		
	Women	-	1.45 (0.90–2.32)
Age	65–74 (Ref)		
	75–84	-	1.34 (0.84–2.13)
	≥85	-	1.26 (0.48–3.28)
Region of residence	Urban (Ref)		
	Rural	-	1.15 (0.72–1.85)
Household type	Single-person (Ref)		
	Multi-person	-	1.66 (0.94–2.92)
Educational attainment	≤Middle school (Ref)		
	≥High school	-	1.13 (0.69–1.86)
Household income	<Standard median (Ref)		
	≥Standard median	-	1.00 (0.59–1.70)
Comorbid Diabetes mellitus	No (Ref)		
	Yes	-	0.65 (0.36–1.18)
Self-rated health	Good (Ref)		
	Poor	-	1.36 (0.78–2.39)
Depressive symptoms	No (Ref)		
	Yes	-	1.55 (0.79–3.03)

OR = Odds Ratio; CI = Confidence Interval; Ref = Reference category; - = not included in the model. ^†^ Estimates from complex sample logistic regression; the three environmental scores were modeled as continuous variables, and odds ratios represent the change in odds per one-point increase. ** *p* < 0.01.

## Data Availability

The data used in this study are publicly available from the Korea Disease Control and Prevention Agency (KDCA) through the Korea Community Health Survey (KCHS) website (https://chs.kdca.go.kr/chs/index.do, accessed on 12 July 2026). Researchers may download the raw data after completing an online application form describing the purpose and intended method of use.

## Supplementary Materials

The following supporting information can be downloaded at: https://www.mdpi.com/article/10.3390/healthcare14182903/s1. Table S1: Associations between individual types of social participation and antihypertensive medication non-adherence; Table S2: Sensitivity analyses of the associations between socio–physical environmental factors and antihypertensive medication non-adherence.
