# Supplementary material for: Social and Physical Environmental Factors Associated with Antihypertensive Medication Non-Adherence Among Older Adults with Hypertension in South Korea: Findings from the 2023 Community Health Survey
Source: Healthcare (Basel). 2026 Sep 8;14(18):2903. doi: 10.3390/healthcare14182903 (PMC13606581; doi:10.3390/healthcare14182903)
Supplement: Supplementary file 1 [file healthcare-14-02903-s001.zip › healthcare-4504037-supplementary.pdf]

healthcare

MDPI

Table S1. Associations between individual types of social participation and antihypertensive medication non-adherence

Type of social participation	aOR (95% CI)
Religious activities	1.27 (0.77–2.09)
Social/fellowship activities	0.97 (0.56–1.69)
Leisure or recreational activities	2.35 (1.46–3.80)**
Charitable organization activities	1.29 (0.58–2.88)

aOR = adjusted odds ratio; CI = confidence interval. Each activity type was entered as a separate binary variable in place of the composite social participation score, with all other covariates identical to Model II (N = 44,253, non-adherence events = 147). The reference category for each activity is non-participation. Estimates were obtained from complex sample logistic regression incorporating the KCHS stratification, clustering, and sampling weights, with estimation restricted to the eligible subpopulation. This analysis was exploratory and involved multiple activity-specific comparisons without adjustment for multiplicity; the results should not be interpreted as confirmed activity-specific effects.
** $p < .01$

Table S2. Sensitivity analyses of the associations between socio-physical environmental factors and antihypertensive medication non-adherence

Analysis	N	Events	Community environment	Social participation	Social contact
Primary analysis (Model II)	44,253	147	0.83 (0.72–0.95)	1.39 (1.09–1.78)	0.85 (0.69–1.04)
Complete responders on community-environment items	42,546	141	0.82 (0.71–0.95)	1.39 (1.08–1.80)	0.80 (0.65–0.97)
90% adherence threshold (<27 days)	44,253	160	0.83 (0.73–0.94)	1.39 (1.11–1.75)	0.85 (0.70–1.03)

aOR = adjusted odds ratio; CI = confidence interval; Events = number of non-adherence events. Values are adjusted odds ratios per one-point increase in each score. All models adjusted for sex, age group, region of residence, household type, educational attainment, household income, comorbid diabetes mellitus, self-rated health, and depressive symptoms. Estimates were obtained from complex sample logistic regression incorporating the KCHS stratification, clustering, and sampling weights, with estimation restricted to the eligible subpopulation.
